# The benefits and risks of bacille Calmette-Guérin vaccination among infants at high risk for both tuberculosis and severe combined immunodeficiency: assessment by Markov model

**DOI:** 10.1186/1471-2431-6-5

**Published:** 2006-03-03

**Authors:** Michael Clark, D William Cameron

**Affiliations:** 1 Division of Infectious Diseases, Department of Medicine, University of Ottawa, General Campus, Room 1805A, 501 Smyth Road, Ottawa, Ontario, Canada, K1H 8L6, USA

## Abstract

**Background:**

Bacille Calmette-Guérin (BCG) vaccine is given to Canadian Aboriginal neonates in selected communities. Severe reactions and deaths associated with BCG have been reported among infants born with immunodeficiency syndromes. The main objective of this study was to estimate threshold values for severe combined immunodeficiency (SCID) incidence, above which BCG is associated with greater risk than benefit.

**Methods:**

A Markov model was developed to simulate the natural histories of tuberculosis (TB) and SCID in children from birth to 14 years. The annual risk of tuberculous infection (ARI) and SCID incidence were varied in analyses. The model compared a scenario of no vaccination to intervention with BCG. Appropriate variability and uncertainty analyses were conducted. Outcomes included TB incidence and quality-adjusted life years (QALYs).

**Results:**

In sensitivity analyses, QALYs were lower among vaccinated infants if the ARI was 0.1% and the rate of SCID was higher than 4.2 per 100,000. Assuming an ARI of 1%, this threshold increased to 41 per 100,000. In uncertainty analyses (Monte Carlo simulations) which assumed an ARI of 0.1%, QALYs were not significantly increased by BCG unless SCID incidence is 0. With this ARI, QALYs were significantly decreased among vaccinated children if SCID incidence exceeds 23 per 100,000. BCG is associated with a significant increase in QALYs if the ARI is 1%, and SCID incidence is below 5 per 100,000.

**Conclusion:**

The possibility that Canadian Aboriginal children are at increased risk for SCID has serious implications for continued BCG use in this population. In this context, enhanced TB Control – including early detection and treatment of infection – may be a safer, more effective alternative.

## Background

Bacille Calmette-Guerin (BCG) is a live, attenuated vaccine derived from *Mycobacterium bovis*. The vaccine provides 65–95% protection against miliary and meningeal tuberculosis (TB) in children vaccinated as neonates [1]. Local and systemic adverse reactions to BCG have been summarized [2,3]. The most severe of these – and fortunately the rarest – is disseminated BCG infection. This complication usually occurs among children with underlying congenital or acquired immunodeficiency disorders [4,5].

BCG use in Canada is confined to high-risk groups, and routine neonatal vaccination programs exist only in TB-endemic Aboriginal (First Nations and Inuit) communities. Since 1982, eight cases of disseminated BCG infection have been reported in this population [6-9]. Five of these infants were diagnosed with congenital immunodeficiency disorders, including four with severe combined immunodeficiency (SCID). Two infants were infected with human immunodeficiency virus (HIV). All of them died either from BCG infection or other complications associated with underlying disease. These events have led to a review of BCG safety issues and policy in Canada.

We developed a state-transition, Markov model to predict the benefits and risks of BCG in Aboriginal infants under varying epidemiologic conditions. There were four objectives in this study: 1) to establish utility values for acute TB states and the state of permanent neurological sequelae following acute tuberculous meningitis; 2) to estimate the future burden of illness in cohorts of children in which a neonatal BCG program is present or absent; 3) to estimate threshold values for the incidence of SCID at birth, above which the decision to give BCG is no longer supported by the model; and 4) to assess the robustness of results to changes in the values for key variables in the model.

## Methods

**Figure 1:**
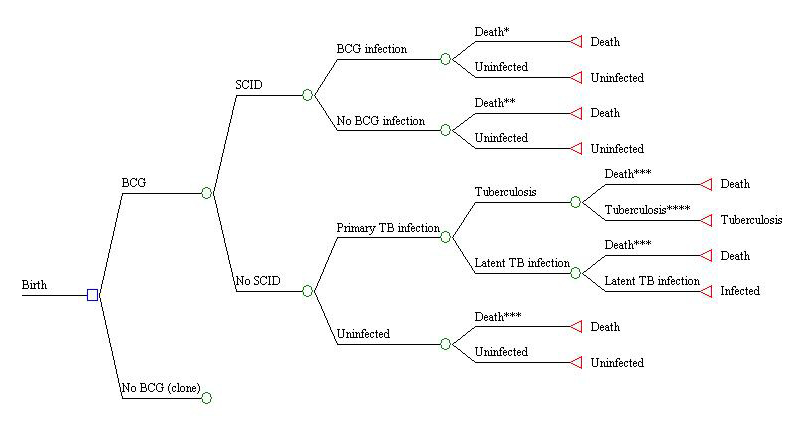
**Diagram of decision model in first Markov cycle (birth to age 6 months)**. * Death due to disseminated BCG infection ** Death due to SCID *** Death due to unrelated causes **** Individual moves to TB disease state (either meningeal, miliary, or other forms of TB) in the next cycle of the model (age 6–12 months).

A Markov model was constructed using DATA 4.0 software (TreeAge Software, Inc., Williamstown, MA). Similar to previous models used to assess BCG [10,11] the model followed cohorts throughout childhood (birth to 14 years). The cycle length was six months, due to several assumptions described below. Analyses were done for theoretical populations experiencing different risks of tuberculous infection, and different risks of SCID in newborns. The model compared a control scenario (no BCG vaccination) to intervention with BCG vaccine. The decision model for the period from birth to age six months is depicted in Figure 1. Model branches and subtrees were identical for both BCG program options, and probabilities for movement into different states affected by vaccination (disseminated BCG infection and TB disease states) were linked between cohorts using expressions of relative risk [12].

**Figure 2:**
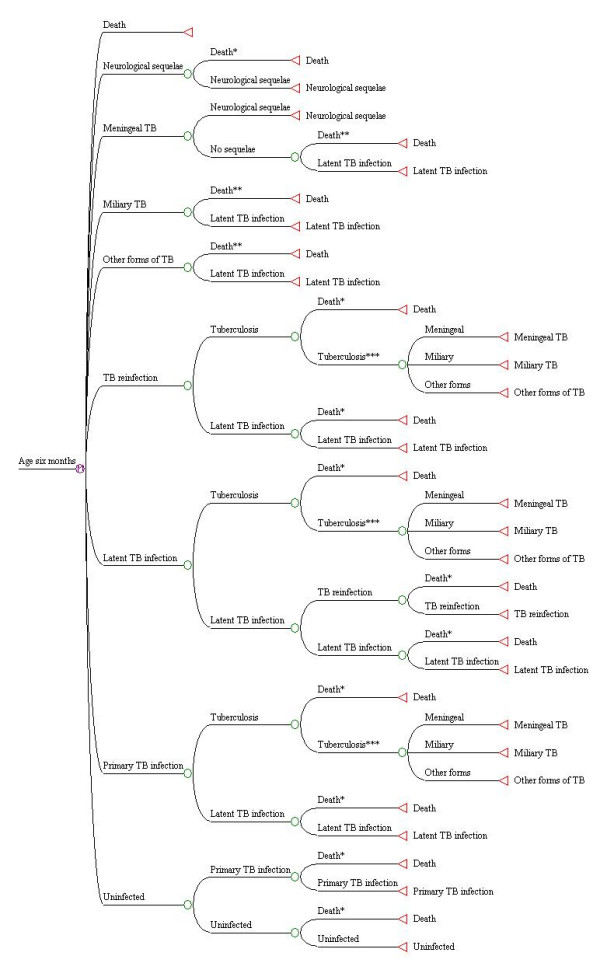
**Diagram of health states in the Markov model beyond age 6 months**. * Death due to unrelated causes ** Death due to acute TB states *** Individual moves to TB disease state in the next Markov cycle.

Health states in the Markov model beyond the age of six months (after the first cycle) are shown in Figure 2 . Uninfected children remain well until the last stage in the model, unless primary tuberculous infection or death due to unrelated causes intervene. Children with primary infection may progress to disease or move into the latent infection state. Children with latent infection may experience exogenous reinfection (exposure to a new strain of *M. tuberculosis*). Reinfected individuals who do not develop disease return once again to the latent infection state. Proportions of uninfected children who experience primary infection, and latently infected children who experience exogenous reinfection, depend on the annual risk of tuberculous infection (the ARI). This represents the overall proportion of the total population infected with *M. tuberculosis *each year.

Children in all three infection states described above may develop TB disease. Among those who develop disease, three states are possible: TB of the central nervous system (CNS) and meninges (ICD-9 013); miliary tuberculosis (ICD-9 018), and non-meningeal, non-miliary TB. The latter state includes all forms of TB disease (including primary and respiratory disease) with ICD-9 codes other than 013 or 018. Children with meningeal TB may develop permanent neurological sequelae. All children with acute TB who do not die or experience disability move into the latent infection state.

Several assumptions were made in the model. First, the theory that BCG protects against primary infection and endogenous reactivation, but not against exogenous reinfection, is applied in the model [13]. The state of SCID lasted one cycle, through the first six months of life. This is based on a review that found that the median age at which infants receive a bone marrow transplant (BMT) is approximately six months [14]. Since SCID is fatal among infants who do not receive a successful BMT [15], infants with this disorder can move into two possible states at age six months: death; or uninfected. Therefore, it is assumed that children who receive a successful BMT experience a normal state of health afterwards [15]. It is also assumed that exposure of SCID patients to tuberculous infection (the risk of infection with *M. tuberculosis*) is nil during the first stage of the model (Figure 1).

The model makes the conventional assumption that once infected with *M. tuberculosis*, an individual remains infected for life [16]. The primary infection and reinfection states are assumed to last six months, as children who develop miliary or meningeal TB generally do so three to six months following initial exposure to TB bacilli [17,18]. Similarly, TB disease states are assumed to last one stage (six months) in the model, due to the availability of short-course, six-month regimens for TB treatment. This is a simplifying assumption: it is recognized that TB cases are often found several months after the onset of disease; and that regimens for treatment of extrapulmonary disease are usually longer than six months [18]. Finally, all forms of TB other than miliary disease and tuberculous meningitis have been grouped into a single state (non-meningeal, non-miliary TB). Evidence for the protective effects of BCG among infants and children has been fairly specific to either all forms of TB disease [19] or miliary and meningeal disease [1].

Probabilities for movement between states in the model are shown in (Table 1). Effectiveness and adverse effects of BCG were estimated from published meta analyses, clinical trials, case series and other studies. Values were based on studies involving Canadian Aboriginal populations, if available [20-24]. The risk of TB disease (all forms) following primary infection was set to 22% (range 14–30%), based on a recent analysis of data from a Canadian First Nations population [23]. This range agrees with other findings in adults [25] and children [26], and approximates the conventionally accepted 10–30% range of risk for disease in children [17]. The risk of tuberculous meningitis following primary infection among 0–4 year olds in the model is 0.88%, within the range of estimates (0.5–1%) reported in the literature [27-31]. Risks of case fatality were estimated using a case-level data set of all reported TB cases (2706) in the Canadian First Nations population (living on and off reserve) between 1990 and 2000. This data set was provided by the Public Health Agency of Canada, Health Canada. Only cases in which TB was considered the cause of death, or TB was not the primary cause but contributed to the death, were considered case fatalities. This information was available for 2550 (94%) of the 2706 First Nations TB cases included in the data set. For meningeal and miliary disease, we calculated proportions of total cases in all age groups who died, due to the very low tally of children with these forms of disease. The proportion of non-meningeal, non-miliary cases who died was estimated using data specific to the 0–14 age group. Ranges for variability and uncertainty analyses were based on 95% confidence limits for these proportions.

Utility values in the model are also shown in (Table 1). Values for the three forms of acute tuberculosis were estimated using hospitalization data from a computerized provincial discharge file provided by Manitoba Health. The median duration of hospitalization was given a utility value of zero, and this time period was subtracted from 182.625 (the average number of days in six months) to obtain a utility value for the six-month acute TB state [40]. Values for variability and uncertainty analyses were obtained in a similar way, using the upper and lower interquartiles for duration of hospitalization.

To estimate a utility value for the state of permanent neurological sequelae following tuberculous meningitis, subjects were recruited to participate in an interview involving the standard gamble technique. During the interview, subjects were offered two alternatives, the first of which was the possibility of perfect health for the remainder of life with a probability of immediate death, and the second of which was living the rest of life with permanent sequelae from tuberculous meningitis. The probability of death was varied between 0 and 1 until the individual was indifferent between the alternatives, and the utility score for the health state in question became one minus that probability [41,42].

A computer tool developed in Excel 2000 software (Microsoft, Inc., Redmond, WA) was used during the interviews. The two alternatives in the standard gamble were shown to the subject, and the probability of death was reflected in a pie chart on the computer screen. Before completing the exercise for the state of permanent sequelae following tuberculous meningitis, two examples were done. The states involved in these examples were blindness in one eye, and then blindness in both eyes. If the participant accepted a higher risk of death (thus having a lower utility score) for blindness in one eye than for blindness in both eyes, it was assumed that the individual had a poor understanding of the exercise and the interview was terminated.

The description of the health state for permanent sequelae following tuberculous meningitis consisted of a brief text describing important elements of the state, followed by descriptors from the Health Utility Index (HUI) Mark III [42]. The brief text was based on a review of 180 patients with tuberculous meningitis in India [34]. It was assumed that neurological sequelae included "moderate residual damage" (hemiparesis, involuntary movements, and substantial mental impairment). Rarer complications such as blindness, deafness, and hypopituitarism [43,44] were not included. A TB medical consultant and a neurologist provided feedback and input in developing the description. During the interview, the health state was described as "Condition X."

Ethical approval for the interview process was sought and obtained from the Ottawa Hospital Research Ethics Board. Signed written consent for participation in the study was obtained from subjects before starting the interview. Volunteers for interviews were recruited from three groups: first-year medical students at the University of Ottawa (year of entrance 2002); employees at Health Canada; and staff at the Department of Social Development and Health, Mohawk Council of Akwesasne. The latter group was included, because the only routine BCG programs in Canada are delivered in First Nations and Inuit communities. Analyses were done to assess the effects of factors such as group, sex, level of education, age, and parental status on utility scores. These data were collected from individuals prior to the interview, and entered into an Access (Microsoft, Inc., Redmond, WA) database. The data were later exported to SPSS^® ^11.0 (SPSS Inc., Chicago, IL) for statistical analyses.

Base case and Markov cohort analyses were completed for four hypothetical cohorts, experiencing different BCG programs and risks of tuberculous infection. Two of the cohorts (unvaccinated controls and those receiving BCG) were exposed to an ARI of 1%, while the other two cohorts were exposed to an ARI of 0.1%. The ARI in a given cohort was converted to the six-month transition probability R using the following expression [45]: R = 1 - (1 - ARI)^0.5^. These analyses were carried out using the base probability and utility values in the model, with the incidence of SCID among newborns set to 0. Outcomes measured in base case analyses were the number of TB cases occurring (meningeal, miliary, and other), and quality-adjusted life years (QALYs) in each cohort. Future QALYs were discounted at a rate of three percent [46]. In Markov cohort analyses, the probability of individuals being in each state in each year of the model was estimated.

Variability was assessed using sensitivity analyses. To evaluate the impact of SCID on the decision to use BCG, the threshold incidence of SCID above which BCG is not supported by the model (above which BCG reduces the number of QALYs) was first calculated assuming an ARI of 0.1%. The robustness of this threshold value was assessed by varying single parameters across plausible ranges in one-way sensitivity analyses. Two-way sensitivity analyses were then carried out on the ARI and SCID incidence at birth, to estimate threshold values across a range of risks for tuberculous infection (0.1 – 1%).

Uncertainties in probability and utility values in the model were assessed using probabilistic Monte Carlo simulation [47,48]. The utility value for neurological sequelae was assigned a normal distribution. Variables estimated from a range of published reports, or a median and interquartile range, were assigned triangular distributions. Empiric probability variables (e.g. TB case fatality estimates) were assigned β distributions [47]. A range of outcomes (mean discounted QALYs and 95% confidence limits) was generated for each of the four cohorts described in the previous section. First, Monte Carlo simulations were done for cohorts in which the risk of SCID is 0. These analyses were then repeated assuming increasing risks of SCID in the population. The risk of SCID was varied from one to 50 per 100,000 births, the approximate risks reported in three European populations [36,49,50] and a North American Aboriginal population [51], respectively. Quality-adjusted life year outcomes in the two cohorts (vaccinated and unvaccinated) were considered significantly different if 95% confidence limits for the two outcomes did not overlap.

**Table 1:** Input data for Markov model.

**Parameter**	**Value (range)**	**Source(s)**
Annual risk of tuberculous infection	(0.001–0.01)	Assumed
Annual rate of decline in the risk of infection	0.14 (0.0–0.14)	23
Relative risk of primary infection if vaccinated^a^	0.39 (0.20–0.43)	20–22, 32
Relative risk of meningeal or miliary TB if vaccinated^a^	0.14 (0.05–0.35)	1,19
Risk of TB following primary infection^a^	0.22 (0.14–0.30)	23, 25, 26
Risk of TB due to reactivation^a^	0.0009 (0.0008–0.001)	23, 25
Risk of TB following exogenous reinfection^a^	0.058 (0.028–0.092)	23, 25
Proportion of TB cases with meningeal TB (0–4 years)	0.040 (0.033–0.047)	33
Proportion of TB cases with meningeal TB (5–14 years)	0.015 (0.006–0.020)	
Risk of neurological sequel from meningeal TB^a^	0.29 (0.13–0.39)	24, 34, 35
Proportion of TB cases with miliary TB (0–4 years)	0.0086 (0.0050–0.012)	33
Proportion of TB cases with miliary TB (5–14 years)	0.0055 (0.0015–0.01)	
Risk of disseminated BCG infection among vaccinated infants born with SCID^b^	0.36 (0.19–0.56)	36
Risk of fatality from meningeal TB^b^	0.091 (0.030–0.15)	Health Canada data
Risk of fatality from miliary TB^b^	0.29 (0.23–0.36)	
Risk of fatality from other forms of TB^b^	0.0013 (0.0013–0.007)	
Risk of fatality, unvaccinated infants with SCID^a^	0.25 (0.19–0.33)	37–39
Risk of fatality, disseminated BCG infection^a^	0.87 (0.8–0.97)	2, 5
Utility value for acute meningeal TB^a^	0.93 (0.81–0.95)	Manitoba hospital data
Utility value for miliary TB^a^	0.92 (0.69–0.97)	
Utility value for other forms of TB^a^	0.98 (0.97–0.98)	
Utility value for neurological sequel following acute meningeal TB^c^	0.43 (0.37–0.49)	Survey

Distributions in Monte-Carlo simulations: a triangular; b β; c normal

## Results

A total of 107 individuals were interviewed in the survey (Table 2). Four interviews were terminated due to lack of comprehension. Mean values for blindness in one eye and blindness in both eyes were 0.76 (95% CI 0.71, 0.80), and 0.59 (95% CI 0.54, 0.65), respectively. The overall mean utility value for neurological sequelae following tuberculous meningitis was 0.43 (95% CI 0.37, 0.49). Mean utility scores did not differ significantly across any of the groups in (Table 2). They varied from 0.40 to 0.47, all within the 95% confidence limits of the overall mean. Age was not a significant predictor of utility scores, according to linear regression analysis (p = 0.36).

**Figure 3:**
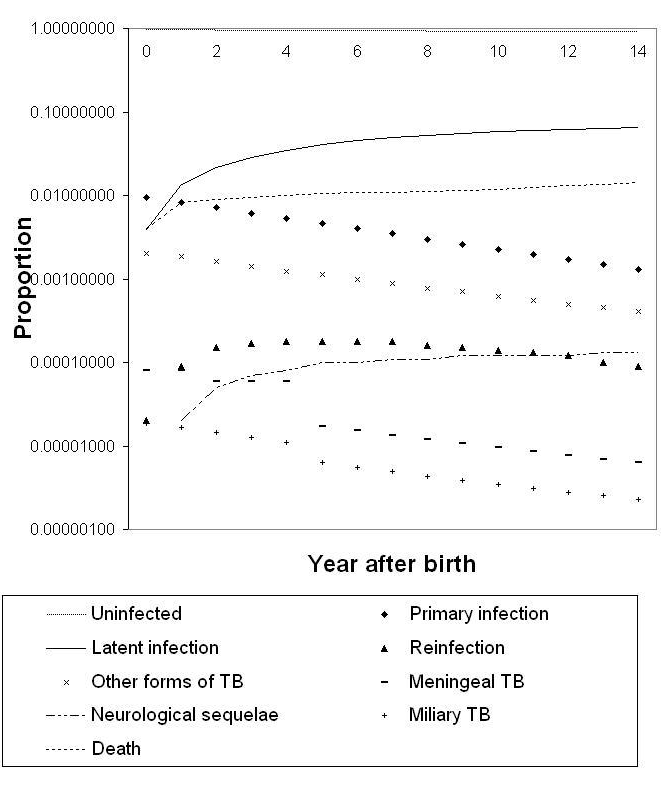
Example of Markov cohort analysis produced by the model, assuming the ARI is 1%, SCID incidence is 0, and no BCG is given.

**Figure 4:**
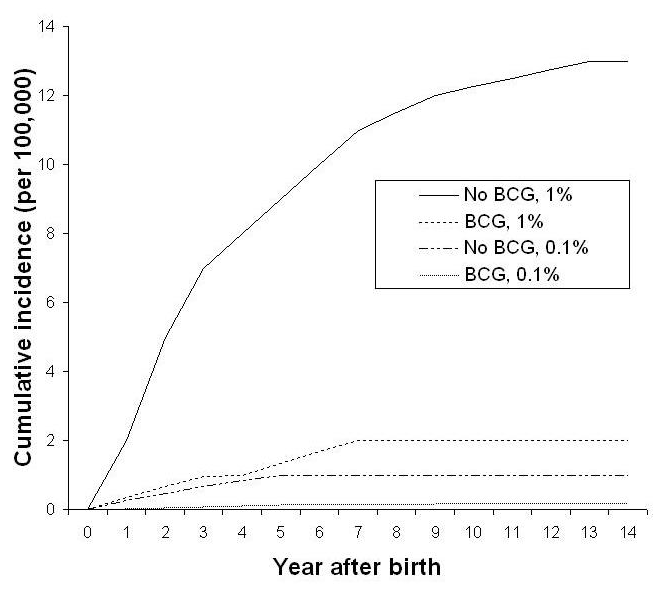
Predicted cumulative number of children living with permanent neurologic sequelae following tuberculous meningitis, by BCG program option and ARI (%).

Markov cohort analyses for a population in which the ARI is 1%, in the absence of BCG vaccine and SCID, are shown in Figure 3. With this ARI, the model predicts that BCG will prevent 961 total TB cases and six deaths in a cohort of 100,000 children, over 15 years (Table 3). In the same cohort, 38 cases of meningeal TB and nine cases of miliary TB are prevented by BCG. The vaccine prevents neurological sequelae in 11 children. Figure 4 depicts the accumulation of children with permanent neurological sequelae following acute tuberculous meningitis. If the ARI is 1% and no BCG is given, a total of 13 cases is predicted in a cohort of 100,000 children over 15 years. The tally is less than one per 100,000 in BCG vaccinated and unvaccinated cohorts, if the ARI is 0.1%.

**Table 2:** Mean utility scores for neurological sequelae following acute meningeal TB, by group, sex, level of education, parental status, and age group

**Group**	**Sample size**	**Mean (95% CI)**
Overall	103	0.43 (0.37, 0.49)
Medical students	37 (36%)	0.46 (0.37, 0.55)
FNIHB employees	34 (33%)	0.42 (0.31, 0.53)
Akwasasne	32 (31%)	0.41 (0.29, 0.53)
Male	30 (29%)	0.42 (0.32, 0.53)
Female	73 (71%)	0.43 (0.36, 0.51)
No university	50 (49%)	0.46 (0.37, 0.55)
University	53 (51%)	0.40 (0.33, 0.48)
No children	50 (49%)	0.44 (0.36, 0.53)
One or more children	53 (51%)	0.42 (0.33, 0.50)
0–34 years old	50 (49%)	0.47 (0.38, 0.55)
35 years or older	53 (51%)	0.40 (0.31, 0.48)

**Table 3:** Estimated TB case tallies and deaths in a birth cohort of 100,000, by ARI and BCG program decision (birth to age 14 years)

**Outcome**	**1% ARI**		**0.1% ARI**	
	**No BCG**	**BCG**	**No BCG**	**BCG**

Tuberculosis (all forms)	1571	610	160	60
Meningeal TB	44.7	6.4	4.5	0.6
Miliary TB	11.3	1.6	1.1	0.2
Neurological sequelae	13.0	2.0	1.0	0.2
TB-related deaths	7.3	1.1	0.7	0.1

**Figure 5:**
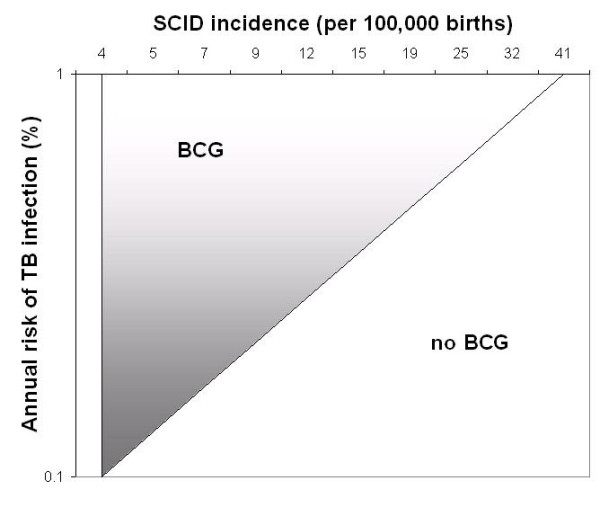
**Two-way sensitivity analysis on the assumed ARI (%) and the incidence of SCID among newborns in the population**. The parameters ARI and SCID incidence were varied across specified ranges to assess sensitivity of the model to these changes. BCG: QALYs significantly higher among vaccinated; No BCG: QALYs significantly higher among unvaccinated

If the ARI is 0.1%, the threshold value for SCID incidence – above which BCG is not supported by the model – is 4.2 per 100,000 births, in sensitivity analyses (Figure 5). This threshold was not altered to more than 8.0 per 100,000, or to less than 2.7 per 100,000, by varying the parameters across their specified ranges in sensitivity analyses. The threshold was most sensitive to the risk of disseminated BCG infection among vaccinated infants born with SCID. Results of the two-way sensitivity analysis on ARI and SCID incidence are shown in Figure 5. If the ARI is increased to 1%, the threshold value for SCID is 41 per 100,000 births.

**Figure 6:**
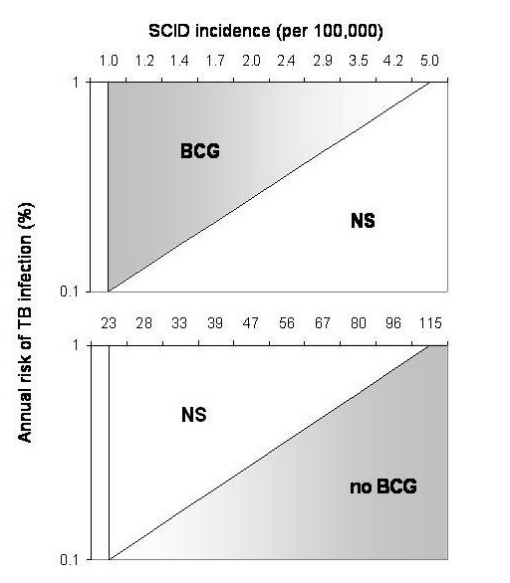
**Uncertainty analysis on the assumed ARI (%) and the incidence of SCID among newborns in the population**. Results of probabilistic Monte Carlo simulation. BCG: QALYs significantly higher among vaccinated; NS: no significant difference in QALYs between cohorts; No BCG: QALYs significantly higher among unvaccinated.

The results of uncertainty analyses (Monte Carlo simulations) are presented in an Appendix . Figure 6 shows threshold values for SCID incidence and the risk of tuberculous infection which affect the decision to give BCG. There are many epidemiologic scenarios in which BCG does not significantly increase or decrease QALYs in cohorts of children, when uncertainties in model parameters are accounted for. If the ARI is 0.1%, estimated QALYs are not significantly increased by BCG unless there is no SCID occurring in the population. If the risk of SCID is 23 per 100,000 births or higher, BCG is associated with a significant reduction in QALYs. With an ARI of 1%, BCG was associated with a significant increase in QALYs if the incidence of SCID was lower than 5 per 100,000.

## Discussion

To our knowledge, this is the first model to date which considers the impact of SCID incidence on the decision to give BCG. Another similar decision model considered the risk of fatal BCG disease, but this risk was fixed at 1 per 10,000,000 vaccinated [10]. The risk among Canadian Aboriginal people is much higher. In a retrospective review of BCG-associated adverse events from Sweden, the implications of immunodeficiency disorders on BCG policy were discussed [52]. Interestingly, these authors recommended that BCG vaccination be confined to high-risk groups and given at age six months, in order to reduce severe disease and deaths among infants with immunodeficiency disorders. In Canada, we face a similar dilemma a decade later.

Results show clear benefits from BCG vaccination when the risk of tuberculous infection is 1% per year. These findings are consistent with other analyses [10,53] and recommendations for the use of BCG in North American populations [54,55]. These benefits become less clear when the ARI is 0.1%. Rates of severe TB disease and deaths are quite low, regardless of the BCG vaccination policy. The threshold for risk of SCID above which BCG is associated with a decrease in QALYs in sensitivity analyses (4.2 per 100,000 births) is only slightly higher than the SCID incidence reported in three European populations [36,49,50]. Results of the study are therefore consistent with recommendations of the International Union against Tuberculosis and Lung Disease and the World Health Organization, which state that BCG discontinuation can be considered in populations with an ARI lower than 0.1% [56,57].

These results have serious implications for populations in which the risk of congenital immunodeficiencies is elevated. SCID is no longer associated with 100% fatality, as it was prior to the availability of bone marrow transplantation [15]. The most important factor in improving the prognosis of an infant with SCID is to diagnose and treat the disease with bone marrow transplantation before overwhelming infections occur [58]. BCG is usually given within days of birth, and no high-throughput screening test for SCID is currently available [59]. The rate of disseminated BCG infection observed among vaccinated First Nations newborns during the 1996–2000 period (20 per 100,000, 95% C.I. 6.2, 68) is more than 40 times higher than rates observed in European populations [60]. Assuming a case fatality rate of 80% [2,5], it can be predicted that 16 per 100,000 infants would die of disseminated BCG infection in a population with this level of risk. Our model estimates that TB-related deaths do not exceed 8 (in a cohort of 100,000 children over 15 years), even if the ARI is 1% and no BCG program is in place.

The sensitivity analyses depicted in Figure 5 may provide useful estimates for the risk neutral decision maker. For those concerned with uncertainties in model parameters, we also conducted probabilistic Monte Carlo simulations. As shown in the Appendix, the magnitude of difference in QALYs between vaccination strategies appears quite small. It would certainty appear much larger for a life-saving intervention in a cohort of cancer patients. However, in the case of BCG we are dealing with an intervention in an entire birth cohort, which may cause or prevent a relatively rare outcome. In this sense, the model accurately reflects what policy-makers must consider when debating the future use of this vaccine, that is, the handful of disease cases or deaths each year affected by its use. In Figure 6, it is apparent that the model does not support either vaccine option for a wide range of ARI and SCID incidence values. However, it may be of interest to decision-makers that BCG is associated with a significant reduction in QALYs for a range of SCID rates and ARI values which may indeed reflect true epidemiologic parameters in Canadian Aboriginal populations.

Several limitations in this study deserve comment. Secondary effects of BCG vaccination due to reduced transmission were not included in the model. Although long-term protection against disease has recently been shown in persons vaccinated during the 1930s [32], there is no evidence that BCG has reduced the risk of TB infection in any population [61]. In the absence of such evidence, estimating the theoretical herd immunity effects of BCG is of little utility [62]. For these reasons, the Markov modeling approach was considered suitable for this study, and transmission was not included in the model.

Although it has been recommended elsewhere that a lifetime horizon be considered in such studies, [46] our model followed a theoretical cohort from birth to age 14 years. This approach was chosen for consistency with earlier modeling studies on BCG, [10,11] and because current evidence suggests BCG protection lasts perhaps 10–15 years [19,63]. Although the prevention of childhood TB and disseminated BCG infection have long-term benefits in terms of life-years gained, BCG has no known impact on any TB-related risk in adulthood, and as such has no impact on outcomes beyond the age of 15 years, or as described above, the risk of morbidity and mortality in the population. Most importantly, the lifetime horizon approach would not have affected the most important outcome in the model, namely those threshold values for SCID incidence which alter the decision to use BCG for a given risk of tuberculous infection in the population.

The risks of BCG infection in children born with congenital immunodeficiencies other than SCID, or among HIV-infected infants, were not considered in the model. Disseminated BCG infection has occurred in an infant with interferon-gamma receptor deficiency, for example [64]. For the purposes of building a model, data on this condition is scarce in comparison to SCID. Health Canada now requires that the HIV status of mothers be known prior to administering BCG to any First Nations newborn. Although errors are possible, quantifying their occurrence in our model would have been tremendously difficult and of little benefit. Adverse events associated with BCG other than disseminated infection, such as adenitis and osteomyelitis, were also excluded from the model.

Perhaps the most important limitation in this and other modeling studies is that conclusions must be based on assumptions made in formulating the model. It is in this context that we can discuss the main strengths of the study. Where possible we have drawn from the experience of Canadian Aboriginal peoples, in an effort to make results as generalizable as possible to the population currently receiving BCG in this country. Primary data collection was undertaken to establish a meaningful utility value for the state of neurological sequelae due to tuberculous meningitis. Finally, appropriate sensitivity analyses and probabilistic Monte Carlo simulations were conducted to account for uncertainties in model parameter estimates and generate plausible ranges for key outcomes.

When BCG was first administered to First Nations infants in the Fort Qu'Appelle Indian Health Unit in Saskatchewan, an incredible 1% of these infants were dying from TB during the first year of life [65]. Death rates were reduced by 80% in vaccinated infants before antitubercular chemotherapy was available [20]. A beneficial effect was also documented among vaccinated infants in Quebec [66], and among American Indians [32]. The epidemiology has changed since these periods. Analyses indicate that the ARI is likely less than 0.1% among First Nations people in British Columbia [23]. A review of safety, efficacy, and epidemiologic data led to the discontinuation of BCG in that population as of June, 2003. Continued use of BCG in Alberta First Nations communities has also recently been questioned [67]. Only one TB-related death was reported among First Nations children between 1990 and 2000. This was a case of congenital TB, which could not have been prevented by BCG in the infant [60]. A further review of treatment outcome data available from Health Canada for the 1997–2002 period revealed no TB-related deaths among First Nations children aged 0–14 years (Public Health Agency of Canada, Health Canada, unpublished data, 2004). Case rates and even deaths may have been higher in the absence of BCG, but the occurrence of eight deaths associated with the vaccine [6-9] provides ample rationale for a discussion on its risks and continued use.

Safer and more effective vaccines for TB prevention may soon be available [68]. One alternative intervention already exists in the form of early detection and treatment of tuberculous infection. The provision of isoniazid is highly effective in reducing the risk of disease [69] and protection may last for up to 30 years [70]. Treatment of infection is generally well tolerated by children [71], and compliance is usually much higher than in adults [72,73]. Considering the safety issues outlined in this report, the best course of action may be the removal of BCG vaccine combined with improvements in TB programming [74]. Such improvements must include early case finding in adults to prevent transmission, and early detection and treatment of infection in children through contact tracing and screening in high-risk communities.

## Conclusion

BCG is an effective vaccine for protecting young children against severe forms of TB. However, in the Canadian context, the occurrence of several deaths associated with the vaccine warrants a review of the relative risks and benefits of its continued use. According to our model, the decision to give BCG vaccine in a population is heavily dependent upon the risk of SCID among newborns. Naturally, these findings assume a setting in which access to health care and bone marrow transplantation services is a reality. There is no doubt that Aboriginal infants and children experience a disproportionate burden of TB when compared to other populations in Canada. However, investing in preventive interventions other than BCG may be more appropriate given the findings of this and other studies. Future research should focus on the development of a new TB vaccine, and on optimizing the delivery of currently available interventions. Efforts to accurately determine the incidence of SCID and the ARI in areas where BCG is still in use would also be valuable, to assist in reviewing BCG programs at the regional level.

## Competing interests

The author(s) declare that they have no competing interests.

## Authors' contributions

Both authors contributed towards the conception and design of the study. Statistical analyses were carried out by MC. The article was drafted by MC and DWC. Both also read and approved the final manuscript.

## Appendix

See Additional File 1.

## Pre-publication history

The pre-publication history for this paper can be accessed here:



## Supplementary Material

Additional file 1 **Appendix – Results of Monte Carlo simulations, assuming different risks of SCID in neonates** * outcome is significantly higher for this decision (95% confidence limits for outcomes among vaccinated and unvaccinated cohorts do not overlap) Click here for file
