# Supplementary material for: The benefits and risks of bacille Calmette-Guérin vaccination among infants at high risk for both tuberculosis and severe combined immunodeficiency: assessment by Markov model
Source: BMC Pediatr. 2006 Mar 3;6:5. doi: 10.1186/1471-2431-6-5 (PMC1458340; doi:10.1186/1471-2431-6-5)
Supplement: Additional file 1 — Appendix – Results of Monte Carlo simulations, assuming different risks of SCID in neonates * outcome is significantly higher for this decision (95% confidence limits for outcomes among vaccinated and unvaccinated cohorts do not overlap) [file 1471-2431-6-5-S1.rtf]

Discounted QALYs and 95% confidence limits	1% ARI	0.1% ARI	
	No BCG	BCG	No BCG	BCG	
Risk of SCID: 0 per 100,000 births	
Mean	11.67284	11.67389*	11.67401	11.67412*	
Lower 95% confidence limit	11.67188	11.67363	11.67392	11.67409	
Upper 95% confidence limit	11.67355	11.67404	11.67408	11.67413	
Risk of SCID: 1 per 100,000 births	
Mean	11.67278	11.67382*	11.67398	11.67406	
Lower 95% confidence limit	11.67174	11.67355	11.67389	11.67403	
Upper 95% confidence limit	11.67349	11.67398	11.67405	11.67408	
Risk of SCID: 25 per 100,000 births	
Mean	11.67197	11.67238	11.67315*	11.67262	
Lower 95% confidence limit	11.67103	11.67195	11.67297	11.67225	
Upper 95% confidence limit	11.67270	11.67274	11.67332	11.67294	
Risk of SCID: 50 per 100,000 births	
Mean	11.67109	11.67089	11.67228*	11.67112	
Lower 95% confidence limit	11.67015	11.67018	11.67195	11.67042	
Upper 95% confidence limit	11.67186	11.67158	11.67259	11.67177	
